# Longitudinal study on the influence of sow and piglet vaccination on seroprevalence of *Salmonella* Typhimurium in rearing pigs and at slaughter in a farrow-to-finish production system

**DOI:** 10.1186/s40813-024-00409-2

**Published:** 2024-12-10

**Authors:** Thies Nicolaisen, Hubertus Vornholz, Monika Köchling, Kathrin Lillie-Jaschniski, Detert Brinkmann, Jörg Vonnahme, Isabel Hennig-Pauka

**Affiliations:** 1 Field Station for Epidemiology Bakum, University of Veterinary Medicine Hannover, Foundation, Büscheler Straße 9, 49456 Bakum, Germany; 2 Viehvermarktung Münsterland eG, Bechtrup 22, 59348 Lüdinghausen, Germany; 3 CEVA Tiergesundheit GmbH, Kanzlerstraße 4, 40472 Düsseldorf, Germany; 4 Fleischhof Rasting GmbH, Eisbachstraße/Am Pannacker, 53340 Meckenheim, Germany; 5 FGS Tierarztpraxis GmbH & Co. KG, Bruchberg 24, 33142 Büren, Germany; 6 Present Address: Institute for Animal Hygiene, Animal Welfare and Farm Animal Behaviour, University of Veterinary Medicine Hannover, Foundation, Bischofsholer Damm 15, Building 116, 30173 Hannover, Germany

**Keywords:** *Salmonella* Choleraesuis, *Salmonella* Typhimurium, Sow vaccination, Piglet vaccination, Sow, Piglet, Seroprevalence, Blood samples, Meat juice

## Abstract

**Background:**

*Salmonella* is widespread in pig husbandry and pork is an important source for human salmonellosis. Surveillance programmes are conducted in many European countries and various management measures are implemented on farm level to control *Salmonella*. Piglet or maternal vaccination can reduce *Salmonella* shedding and lower the likelihood of piglet infection. Proper management of risk factors can help to maintain low infection pressure. The aim of the study was to evaluate the effect of sow vaccination and piglet vaccination on *Salmonella* seroprevalence at slaughter.

**Results:**

Different vaccination strategies were evaluated for their effect on seroprevalences in nursery (serum) and slaughter pigs (meat juice) in a farrow-to-finish production chain tested positive for *Salmonella* Typhimurium (ST). Antibody levels of four piglet groups from one rearing farm and of pigs from four downstream fattening farms were measured by *Salmonella* LPS-ELISA in a longitudinal study (UNVAC: no vaccination against *Salmonella*; PIGVAC: piglets vaccinated twice with an attenuated *Salmonella* Cholerasuis (SC) live vaccine; SOWVAC-1: piglets born from sows vaccinated twice before farrowing with attenuated ST live vaccine; SOWVAC-2: Piglets from vaccinated sows (ST) which had been vaccinated twice already as a piglet (ST). Results revealed significantly lower ELISA optical density (OD) values (p < 0.05) and fewer serological positive piglets (OD > 40) from groups PIGVAC, SOWVAC-1 and SOWVAC-2 compared to group UNVAC at the end of rearing period. Summarizing results from pigs of all fattening farms revealed that pigs from group PIGVAC had significantly lower ELISA OD values in meat juice samples than all other groups (p < 0.05).

**Conclusion:**

Piglet (SC) and sow vaccination (ST) led to significant reduction in detectable antibodies in a ST positive production chain and thus to reduced likelihood of infection during rearing. The results reflect that vaccination with a live attenuated SC vaccine resulted in cross-protection against ST without producing antibodies detectable by standard *Salmonella* LPS-ELISA. Summarizing all fattening farms, piglet vaccination reduced seroprevalence at the time of slaughter. In conclusion, sow and piglet vaccination with attenuated live vaccines against *Salmonella* are good instruments to reduce the infection pressure in the rearing period but need additional management measures to show effect on seroprevalence at slaughter.

## Background

Infections with non-typhoidal *Salmonella* were the second most common human gastrointestinal zoonosis in the European Union in 2022 [1]. Non-typhoidal salmonellosis in humans usually causes severe, self-limiting enterocolitis with fever, nausea, vomiting, abdominal cramps, and diarrhea [2, 3]. Severe disease courses occur primarily in immunocompromised groups and require targeted therapy [2]. Pork and its products play an important role in foodborne outbreaks of salmonellosis in humans [1]. *Salmonella* is widely distributed in swine. Infection with *Salmonella* Typhimurium (ST) in pigs usually results in only subclinical disease and often remains undetected. Pigs shedding *Salmonella* at the time of slaughter can contaminate carcasses during the slaughter process and thus *Salmonella* enters the food chain [4–6].

Serovars most often found on pig and its products are ST, the monophasic variant of ST and *Salmonella* Derby. These three serotypes are also among the top five serovars responsible for non-typhoidal salmonellosis in humans.

ST and its monophasic variant were responsible for 17.4% of non-typhoidal salmonellosis cases in humans in Europe in 2022 [1]. Therefore, many European countries have control programmes aimed at either completely eradicating salmonellosis in pigs (e.g., Sweden, Norway, Finland) or reducing the incidence of infection and thus the risk of transmission into the food chain (e.g., Germany) [7]. The "QS Qualität und Sicherheit GmbH” (QS) has been carrying out the national *Salmonella* monitoring in Germany since 2002. For this purpose, 15 samples are quarterly (60 samples per year) either taken at the slaughterhouse (meat juice samples) or within 14 days before slaughter (blood samples). This maximum number of random samples in the *Salmonella* monitoring applies to all fattening farms that sell more than 200 pigs for slaughter per year. These samples are serologically tested for antibodies against *Salmonella* by a commercial LPS-ELISA test admitted for this purpose. The defined cut-off value is set at percentage positivity of 40% based on the quotient of optical density (OD) values of the sample and the positive control corrected by the OD of the negative control. All samples with OD ≥ 40% are considered positive in the German *Salmonella* monitoring. Farms are categorized according to their *Salmonella* status by the proportion of positive reactors in category I (0–20% positive samples), category II (21–40% positive samples), and category III (> 40% positive samples). Third category farms must implement *Salmonella* control measures and may be subject to financial penalties. Many animal and environmental related risk factors influence the *Salmonella* seroprevalence of pigs at the slaughterhouse [8–10]. Knowledge of *Salmonella* serotypes present on farm is valuable to select appropriate control measures, because different *Salmonella* serotypes may be associated with either more animal-related or more environmental-related risk factors, and thus different control measures may lead to varying results [11]. Successful herd sanitation can only be achieved if all risk factors are identified, considered and managed properly. Vaccination against *Salmonella* can be one effective tool to supplement other control measures leading to significant reduction of *Salmonella* infection pressure [12–15]. Studies suggest that not only vaccination of piglets themselves has a positive effect on *Salmonella* antibody detection rates in downstream production stages up to slaughter [16, 17], but also vaccination of sows [13]. Sows are known to harbour the same *Salmonella* serotypes to those of their suckling piglets [18]. Therefore, a vaccination strategy that significantly reduces *Salmonella* excretion of the sow, could lead to fewer infected suckling piglets with a beneficial effect on the entire downstream production stages.

The objectives of this study were assessment of effects of piglet vaccination against *Salmonella* Choleraesuis (SC) and of two different protocols of sow vaccination against ST on the seroprevalence of piglets of a farm positive to Salmonella Typhimurium (ST) at the end of rearing and of fattening pigs at the time of slaughter.

## Material and methods

### Animals and vaccination schemes

The longitudinal study was conducted in a farrow-to-finish production chain consisting of a piglet producer with 1000 reproductive sows and breeding its own replacement gilts, a piglet rearing farm (4000 piglet rearing places) and four fattening farms (fattener 1: 1650 fattening places; fattener 2: 1980 fattening places; fattener 3: 1490 fattening places; fattener 4: 360 fattening places), in western Germany between April 2019 and November 2021. All farms (piglet producing farm, piglet rearing farm, fattening farms) involved belonged to different owners. However, fix cooperative trading relationships existed among the farmers: The piglet rearing farm exclusively bought the piglets after weaning from the piglet producing farm and the four fattening farms bought pigs exclusively from the piglet rearing farm. The piglet producing farm used a weekly batch management with 4 weeks of suckling. Piglets were vaccinated against Porcine Circovirus 2, Porcine Reproductive and Respiratory Syndrome Virus (PRRSV) and Influenza A Virus. During the first period of the study, *Salmonella* Typhimurium and *Salmonella* Choleraesuis were detected during necropsy of wasting nursery pigs. *Salmonella* Typhimurium was also detected in environmental samples taken on farm. At the start of the study in April 2019 the 4 fattening farms were categorized in category 2 (three fattening farms) and 3 (one fattening farm) by the mandatory serological monitoring. This longitudinal study involved four different experimental groups that consecutively underwent the experimental design. The four groups were composed as follows:

- Group UNVAC contained unvaccinated piglets born from six farrowing groups of unvaccinated sows included between March and October 2019.
- Group PIGVAC included piglets from seven consecutive batches vaccinated twice against SC with a conventional attenuated live vaccine (Suisaloral®, Ceva Tiergesundheits GmbH, Düsseldorf, Germany). This vaccine was used, because SC was cultured from wasting nursery pigs during necropsy in a period of bad performance in 2019. Piglets were vaccinated orally at 14 days of life and subcutaneously at 6 weeks of life on the piglet rearing farm. The first group was vaccinated in November 2019.
- Group SOWVAC-1 consisted of unvaccinated piglets that were born within a three-month period from three farrowing groups of sows that were vaccinated twice subcutaneously against ST with a conventional live attenuated *Salmonella* Typhimurium (ST) vaccine (Salmoporc®, Ceva Tiergesundheits GmbH, Düsseldorf, Germany) six and three weeks prior to farrowing. First sows were vaccinated in October 2019.
- Group SOWVAC-2 consisted of unvaccinated pigs from five farrowing groups within a six-month-period, whose mothers had been vaccinated already as suckling piglets: 3rd week of life (oral), 6th week of life (subcutaneous), 160th day of life (subcutaneous) and 3 weeks before farrowing (subcutaneous) against ST (Salmoporc®, Ceva Tiergesundheit GmbH, Düsseldorf, Germany). First group of animals belonging to that group were born in November 2020, as the oral vaccination of future gilts already had started with the first sampling of the nursery in November 2018.

### Target variables, sampling schemes and ELISA test

The target variables ELISA OD percentage positivity (OD%) (quantitative) and proportions of serological positive animals (defined as ELISA OD ≥ 40%) were examined and compared between the four experimental groups at two sampling time points.

The first time point for taking blood samples in pigs randomly selected out of a group was at the end of rearing at 11 weeks of age immediately before transfer to the fattening farm (UNVAC: n = 149; PIGVAC: n = 100; SOWVAC-1: n = 61; SOWVAC-2: n = 151). Blood samples at the end of piglet rearing were taken as part of the farm’s *Salmonella* control plan and *Salmonella* sanitation process. The blood samples were taken by the herd veterinarian. Meat juice samples were taken at slaughter as part of the *Salmonella* monitoring of QS. The meat juice samples were analysed by LPS-ELISA for antibodies against *Salmonella*. As the slaughter pigs could be allocated to the four groups, these samples were used for data collection in the present study (UNVAC: n = 644; PIGVAC: n = 138; SOWVAC-1: n = 72; SOWVAC-2: n = 269).

### Preliminary examinations

To establish the status quo about *Salmonella* contamination before beginning of the herd sanitation, preliminary investigations were conducted in all three sites of the production chain. These investigations included environmental samples (sock and/or wipe swabs) and blood or meat juice samples for detection of antibodies against Salmonella (serogroups B, C1 and D) by ELISA.

Antibodies in serum and meat juice were analysed by PrioCHECK® Salmonella Ab porcine 2.0 (Prionics Deutschland GmbH, Planegg-Martinsried, Deutschland) according to the manufacturer´s instructions.

### Statistical analysis

Number of serological positive animals (positive ≥ OD 40%) and ELISA OD% levels were evaluated as representative variables for the amount and affinity of *Salmonella* antibodies. Statistical evaluations were performed with Excel program, version 2010 (Microsoft Corporation, Albuquerque, USA) and SAS® statistical program, version 9.4 (SAS Institute, Cary, NC, USA). ELISA OD% values were not normally distributed according to the results in the Shapiro–Wilk test and no variance homogeneity existed between vaccination groups (Levene's test, p < 0.0001). Therefore, the Kruskal–Wallis test was used as a parameter-free method for the analysis. Group comparisons were performed with Wilcoxon and Kruskal–Wallis test (different vaccination status, different fattening farms). Frequencies were compared using Fisher`s Exact test. Simple Logistic regression analyses (Wald test) were performed to assess the effect of vaccination and fattening farm as well as their interaction on positive or negative serological findings according to the ELISA OD%-cut off. The logistic regression analyses were performed as main effect binary logit models with two classification variables (vaccination and fattening farm) and their interactions and the Fischer score optimization method as an iterative estimation method. The significance level was set at p < 0.05.

## Results

### Preliminary examinations

#### Piglet producing farm

Preliminary investigations in 2018 at the piglet producer revealed a low detection rate of *Salmonella* Typhimurium in the environment by cultural diagnostic of sock swabs and surface wipes taken in the environment of pigs of all production stages and on the corridors (2% samples positive (1/48)). The positive swab was detected in a farrowing room with sows and piglets 3.5 weeks after farrowing. A critical point in terms of *Salmonella* transmission risk on the farm was insufficient cleaning and disinfection of a central corridor that was used for driving piglets to the transport vehicle after weaning.

#### Piglet rearing farm

Preliminary examinations of environmental samples in cleaned and disinfected compartments of the rearing farm regularly yielded positive *Salmonella* Typhimurium results by bacteriological culture (Table 1). Therefore, an improvement of cleaning and disinfection measures was necessary and implemented on the farm. Vaccination of sows before farrowing at the piglet producing farm started in October 2019 after cleaning and disinfectant measures were optimised and all environmental samples were negative. Cleaning and disinfection measures in the rearing farm were controlled and optimized continuously during the project.

**Table 1:** *Salmonella* positive environmental samples in the cleaned and disinfected piglet rearing farm in chronological order

	Number of samples	Positive bacteriological tests	Proportion of positive bacteriological tests (%)
November 2018	22	9	41
December 2018	21	3	14
June 2019	14	7	50
July 2019	11	0	0
July 2019, incl. drinking water pipes	15	0	0
September 2019	22	10	45
October 2019	27	0	0
November 2019	15	0	0
March 2020	11	2	18
May 2021 incl. transportation vehicle	15	1	7

#### Fattening farms

Before pigs from groups PIGVAC, SOWVAC-1 and SOWVAC-2 were introduced into the fattening farms, environmental samples were taken at random in all four fattening farms (in total n = 42, sock and wipe swabs) also in cleaned and disinfected fattening compartments. All samples were negative, i.e., no *Salmonella* could be detected by culture.

### Study results

#### Nursery pigs

**Fig. 1:**
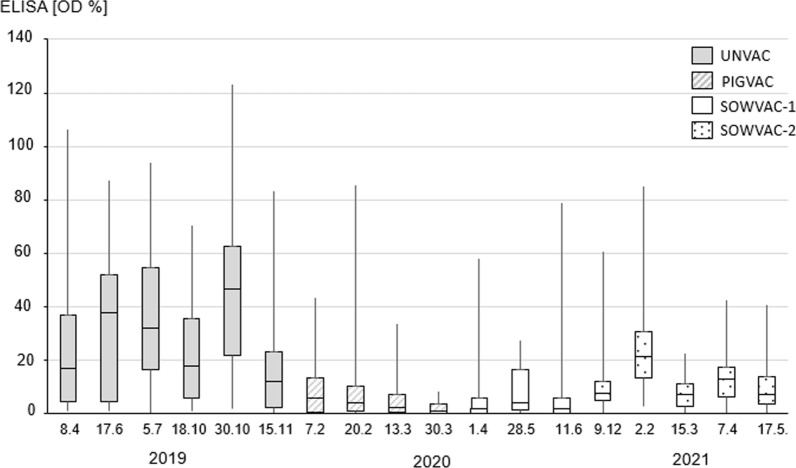
Box plot representation of optical densities measured by ELISA in serum samples from piglets at the end of rearing. Upper and lower whiskers indicate maximum and minimum. Median is indicated in the interquartile range shown as box. The vaccination status of the rearing piglets, which changed during the project, is represented by the different box filling and explained in the legend. On the X-axis, the data of the sample collections are shown, in which at least 15 and a maximum of 48 rearing piglets were sampled

**Fig. 2:**
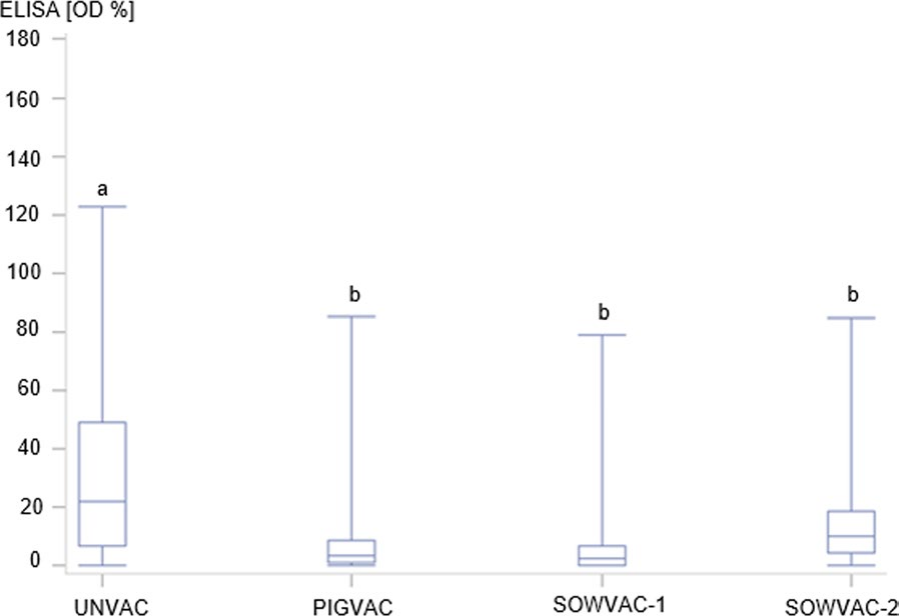
Distribution of ELISA OD values in pigs with different vaccination status at the end of rearing. Upper and lower whiskers of the box plot indicate maximum and minimum. Median is indicated in the interquartile range shown as box. The vaccination status of the rearing piglets is displayed on the X-axis (UNVAC, PIGVAC, SOWVAC-1, SOWVAC-2). Different letters mark significant differences (p < 0.05)

During the project, both a reduction in the OD% measured in the ELISA as an indication of the amount and affinity of salmonella-specific antibodies (p < 0.05) and a decrease in the proportion of serologically positive animals (p < 0.05) were achieved in the piglet rearing farm (Figs. 1 and 2).

The ELISA OD% values of the piglet group UNVAC (mean: 30.8; SD ± 27.7) were significantly higher (p < 0.05) than the values of the groups PIGVAC (mean: 8.5; SD ± 13.5), SOWVAC-1 (mean: 7.4; SD ± 13.4), as well as SOWVAC-2 (mean: 13.7; SD ± 13.2). ELISA OD% values of PIGVAC and SOWVAC-1 groups were not significantly different from each other (p > 0.05), but both were significantly lower compared to those of SOWVAC-2 group (p < 0.05). The results are also presented in Figs. 1 and 2.

**Fig. 3:**
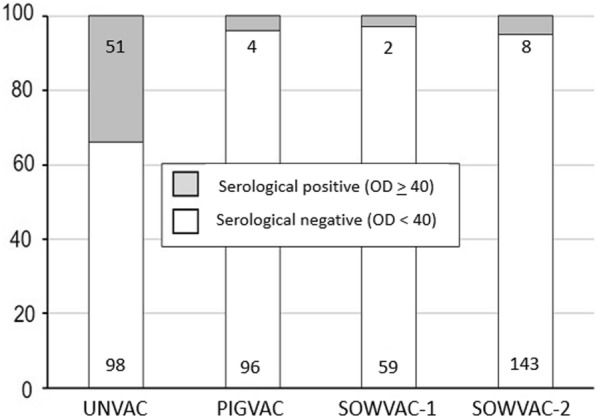
Proportion of seroreagents (%) with different vaccination status (UNVAC; PIGVAC; SOWVAC-1; SOWVAC-2) at the end of rearing. The number of samples is indicated in the columns (serological positive: upper row; serological negative: bottom row)

The distribution of positive and negative samples in the experimental groups were as follows and are illustrated in Fig. 3: UNVAC (positive samples: n = 51; negative samples: n = 98), PIGVAC (positive samples: n = 4; negative samples: n = 96); SOWVAC-1 (positive samples: n = 2; negative samples: 59); SOWVAC-2 (positive samples: n = 8, negative samples n = 143). A frequency comparison of positive animals with different vaccination status revealed a significant influence of vaccination after evaluation with the Fisher exact test in an eight-field table (p < 0.05).

Logistic regression calculations were used to examine the influence of vaccination status on the serological status of the individual animal (positive/negative). Subsequently, a significant influence of the vaccination status was found by the overall Wald test (p < 0.0001).

The vaccination status (PIGVAC, SOWVAC-1, SOWVAC-2) had a significant influence on the serological status compared to the UNVAC group. The calculated odds ratios can be interpreted as follows: PIGVAC piglets were 12.5-fold more likely, SOWVAC-1 piglets were 15-fold more likely, and SOWVAC-2 piglets were ninefold more likely to be serologically negative at the end of rearing compared to UNVAC piglets (Table 2).

**Table 2:** Results of the logistic regression model with target variable negative serological finding at the end of nursery period

Predictor	OR	OR 95% confidence interval	Wald Chi square	p-value
UNVAC (Ref.)	1			
PIGVAC	12.5	4.3–35.7	22.0	< 0.0001
SOWVAC-1	15.4	3.6–66.7	13.6	0.0002
SOWVAC-2	9.26	4.2–20.4	30.7	< 0.0001

UNVAC—no vaccination against *Salmonella*, PIGVAC—piglets vaccinated twice with an attenuated Salmonella Cholerasuis (SC) live vaccine, SOWVAC-1—piglets born from sows vaccinated twice before farrowing with attenuated ST live vaccine, SOWVAC-2—Piglets from vaccinated sows (ST) which had been vaccinated twice already as a piglet (ST)

#### Fattening pigs

**Fig. 4:**
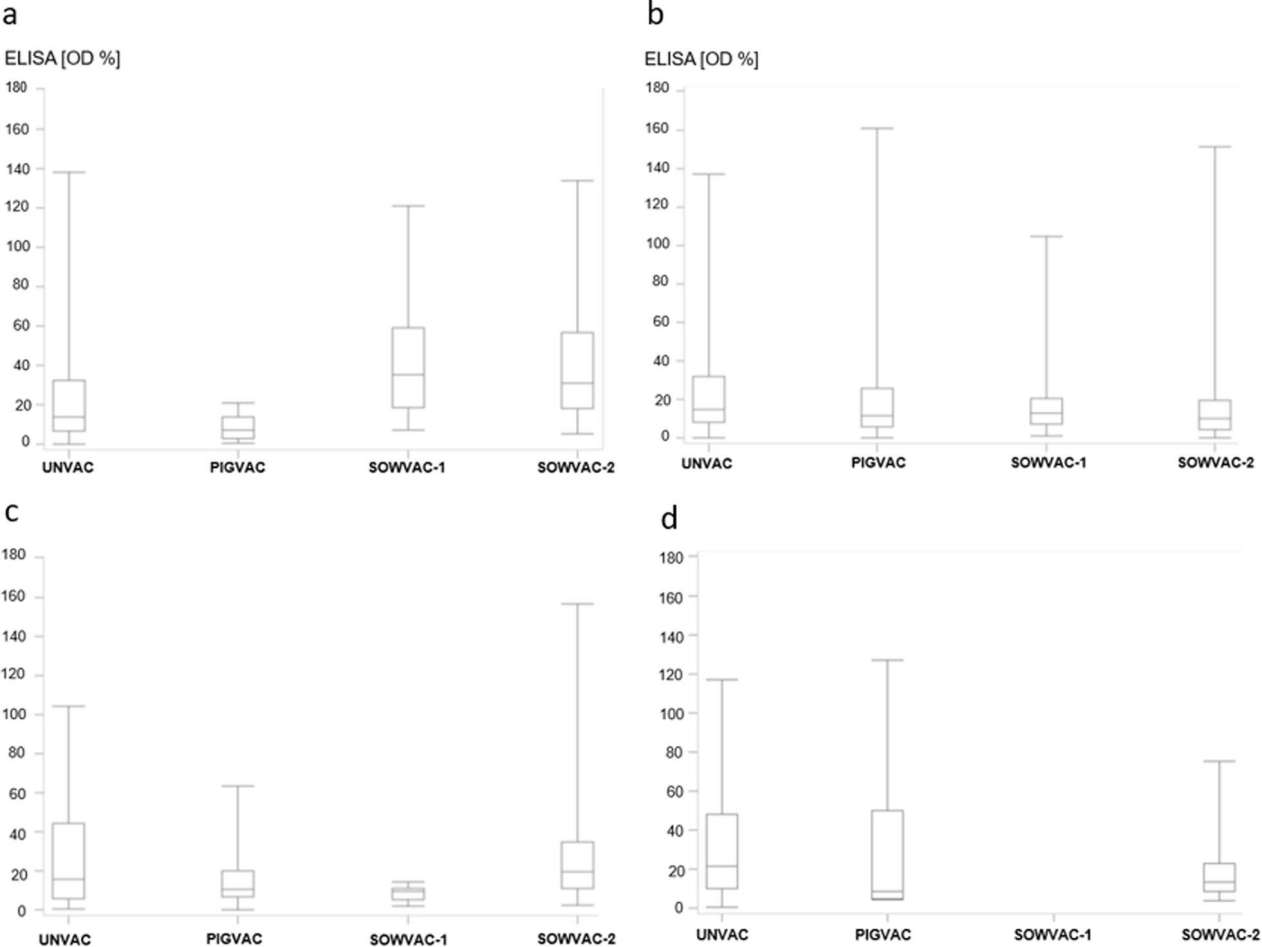
**a**–**d** Distribution of ELISA OD values (meat juice) in slaughter pigs with different vaccination status of the four fattening farms. Upper and lower whiskers of the box plots indicate maximum and minimum. Medians are indicated in the interquartile ranges shown as boxes. Vaccination status of slaughter pigs is displayed on the X-axis. Sample size (n) for the different groups (mean and SD in brackets): **a** Fattener 1: UNVAC: n = 155 (26.2 ± 30.2); PIGVAC: n = 11 (8.4 ± 6.7); SOWVAC-1: n = 26 (45.7 ± 34.5); SOWVAC-2: n = 62 (40.2 ± 31.0); **b** Fattener 2: UNVAC: n = 309 (27.0 ± 25.6); PIGVAC: n = 72 (24.5 ± 33.2); SOWVAC-1: n = 40 (21.7 ± 25.3); SOWVAC-2: n = 66 (16.6 ± 22.3); **c** Fattener 3: UNVAC: n = 96 (28.9 ± 30.0); PIGVAC: n = 48 (14.6 ± 12.0); SOWVAC-1: n = 6 (8.6 ± 4.5); SOWVAC-2: n = 70 (29.4 ± 31.7) **d** Fattener 4: UNVAC: n = 84 (33.3 ± 32.2); PIGVAC: n = 7 (31.0 ± 45.3); SOWVAC-1: no samples; SOWVAC-2: n = 56 (17.1 ± 13.7)

The results for the four fattening farms are shown in Fig. 4a–d. Large differences were detected between the different fattening farms. No consistent trend could be identified in the course of the study.

Evaluation of total data of slaughter pigs with different vaccination status irrespective of farm revealed significantly lower (p < 0.05) ELISA-OD% values for pigs in the PIGVAC group (n = 138, mean and SD: 20.1 ± 27.4) compared to all other groups (UNVAC: n = 644 [mean and SD: 27.9 ± 30.2]; SOWVAC-1: n = 72 [mean and SD: 29.3 ± 30.6]; SOWVAC-2: n = 269 [mean and SD: 25.7 ± 27.7). ELISA OD% values for the SOWVAC-1 and SOWVAC-2 groups were not significantly different from the UNVAC group (p > 0.05).

**Fig. 5:**
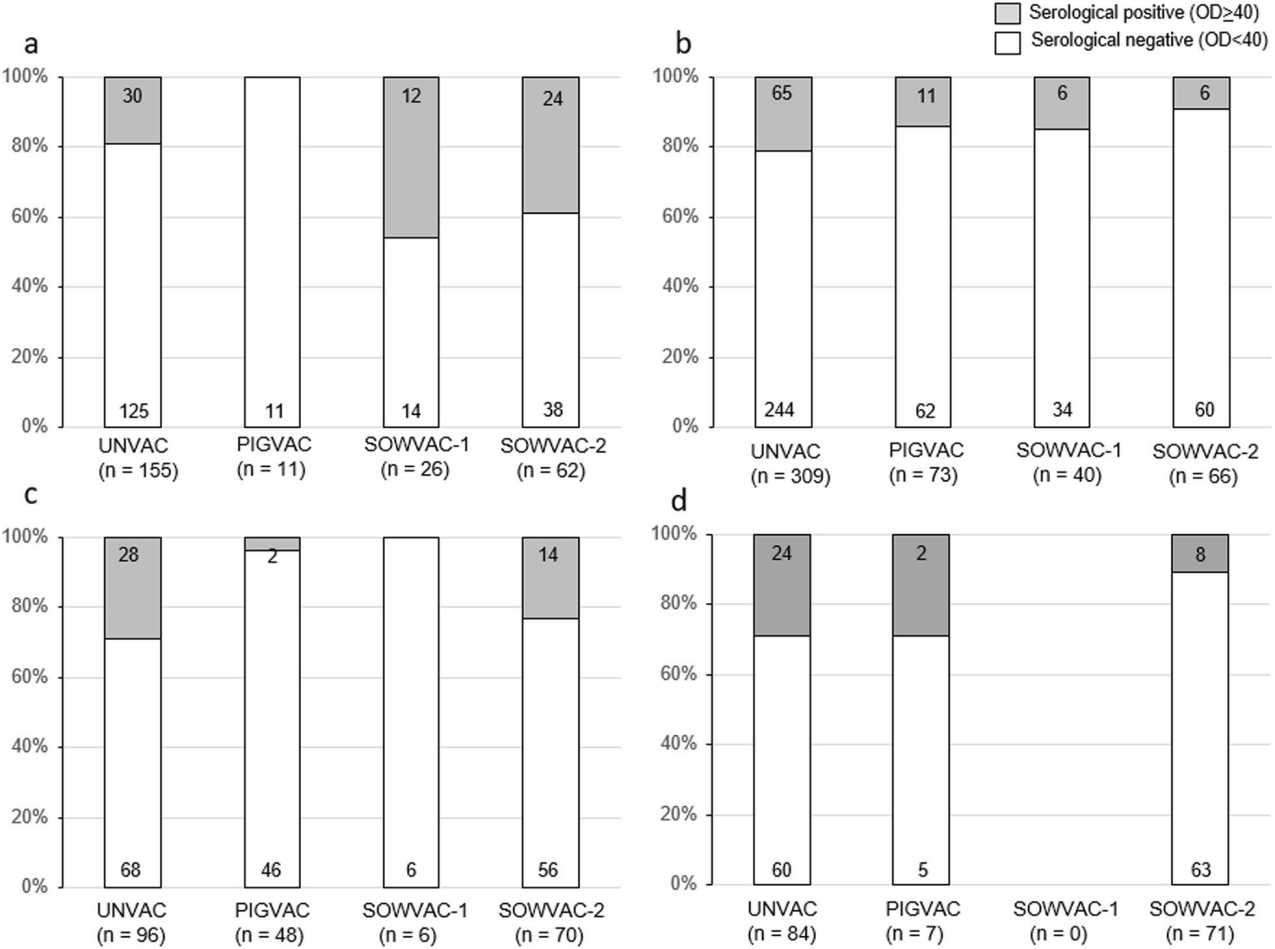
**a**–**d** Proportions of seroreagents (%) in meat juice with different vaccination status in fattening farms 1–4. Sample numbers are displayed in the columns

The proportions of seroreagents in meat juice samples of slaughter pigs of the different fattening farms are shown in Fig. 5a–d. Frequency comparisons of positive slaughter pigs with different vaccination status showed a significant influence of farm (p < 0.05) but not of vaccination status (p > 0.05) for the fattening farms using logistic regression by Wald test (Table 3). The interaction between fattening farm and vaccination status was significant (p < 0.05). Individual evaluations of vaccination status and fattening farms showed that animals in the UNVAC group and fattening farm 1 are responsible for these significant effects (Table 3).

**Table 3:** Selected results of the logistic regression model/Wald test with target variable negative serological finding in meat juice

Predictor	Wald Chi square	p-value
Farm	19.9	0.0002
Vaccination group	3.5	0.33
Farm x vaccination group	29.6	0.0003
Farm 1 (Ref. farm 5)	17.4	< 0.0001
Farm 2 (Ref.: farm 5)	5.2	0.03
Farm 1 × UNVAC (Ref.: farm 5 × SOWVAC-2	18.9	< 0.0001

UNVAC—no vaccination against Salmonella, SOWVAC-2—Piglets from vaccinated sows (ST) which had been vaccinated twice already as a piglet (ST)

## Discussion

This study accompanied the *Salmonella* sanitation process of a farrow-to-finish production chain over a period of approximately two years. The longitudinal effect of an optimized management and different vaccination schemes on the *Salmonella* seroprevalence of pigs at the end of rearing and at slaughter was investigated. Therefore, no parallel groups were possible in this longitudinal study and comparisons were made between samples taken at different time points. It can be assumed that many factors on all participating farms and in their environment that have an influence on seroprevalence could have changed during the study (e.g., differences in the thoroughness of cleaning and disinfection between the batches). This leads to a possible overlap of various unknown effects, which might be partly responsible for the significant differences found. However, other longitudinal studies have already shown a positive long-term effect of vaccination of sows and piglets with a live-attenuated ST vaccine on the *Salmonella* load in the animal environment [19, 20]. Different vaccination strategies were implemented next to measures aimed at optimizing cleaning and disinfection management in the pig production chain (sow farm, rearing farm and linked fattening farms) that participated in this study. The cleaning and disinfection protocols of the piglet producer and the piglet rearing farm were continuously reviewed and optimized before our study started. In addition, a risk assessment was carried out in the farrowing farm and nursery, in which critical points regarding *Salmonella* control were uncovered and thus possible sources of infection of the piglets were eliminated. By improving these identified weak points, the environmental pressure in farrowing and thus a reduced risk to wean already infected piglets, would allow to move piglets with a lower *Salmonella* load into the rearing farm. This could already have had a positive impact on *Salmonella* prevalence in nursery. Additionally, an optimized cleaning and disinfection regime in the downstream piglet rearing farm, which effectively eliminates residual *Salmonella* flora as a potential source of infection, can enhance this effect. It was shown before that the probability of contamination with *Salmonella* in previously *Salmonella* positive rooms was significantly reduced after improved cleaning and disinfection management compared to rooms without improved concepts [21]. Additionally, there is evidence that vaccinated piglets excrete lower amounts of *Salmonella* [17, 22] and therefore recontamination of these rooms should be lower. If unvaccinated piglets are subsequently reintroduced, environmental contamination by excretion may increase followed by an increase of seroreagents over time, if the residual bacteria have not been fully eliminated. Depending on the risk of recontamination during the rearing and growing phase, sow vaccination alone can help to reduce *Salmonella* prevalence in linked fattening farms. This was demonstrated in a study in which lower levels of *Salmonella* excretion in downstream finishing barns were found when sows were vaccinated against ST compared to fattening pigs of unvaccinated sows [20]. Thus, the results of the different experimental groups described in our study cannot be considered independent of each other. It can be assumed that the different groups influenced each other in this longitudinal approach. The divergent results for the individual fattening farms in our study reflect the high impact of appropriate management measures besides vaccination of piglets entering the fattening farms. These management factors are diverse and include among others one-directional pig flow and avoiding mixing of pigs, pest control, cleaning and disinfection, manure management, feed storage and feed additives [23].

### Piglet rearing

The repeated detection of positive environmental sock and swab samples in the cleaned and disinfected rooms in the piglet rearing farm during the investigation period before the start of the vaccination trial, are a clear indication for inadequate hygiene management. This harbored the risk of an early contact to *Salmonella* by piglets born from vaccinated sows during the immunological gap, short after entering the nursery. Due to that it would be impossible to serologically see an effect of sow vaccination, neither end of nursery, nor end of fattening. The first weeks after weaning are considered as a high-risk period for infection with *Salmonella* due to piglets` low levels of *Salmonella*-specific antibodies in seropositive breeding farms [24]. Therefore, vaccination of sows before farrowing was not started in our study until the environmental samples in the piglet rearing farm were all negative after cleaning and disinfection. Despite being able to find rooms positive in October 2019 and November 2019, allowing to start the vaccination trial, later during the trial *Salmonella* Typhimurium was repeatedly detected from environmental samples in cleaned and disinfected rooms again. Based on these findings, it became apparent that different levels of contamination occurred over time. The loads could have been caused on the one hand by entry of carrier animals and increased excretion of *Salmonella*, and on the other hand by inadequate elimination of the pathogens during cleaning and disinfection measures. *Salmonella* sustains for up to 50 months in the environment, e.g. in dust, and up to 24 weeks in manure [25, 26]. Therefore, reinfection by contact with walkways or rooms that were not thoroughly cleaned is a high risk within *Salmonella* control programs.

The serological analysis at the end of rearing showed significant lower mean OD-% values and less positive animals in all treatment groups compared to the non-treated groups before the start of vaccination. Antibodies detected at this first sampling (end of nursery), are most likely linked to direct contact to *Salmonella* either from the environment or due to early infected piglets carrying *Salmonella* into the nursey. It has been shown, that maternally derived antibodies (MDA) generally decline until the end of nursery [27]. These MDA only partially protect against infection, leading to seropositive animals at weaning, already possibly being infected with *Salmonella* [18, 24]. The seroconversion after infection in nursery can vary a lot, depending on the timepoint of infection and the dynamic of infection within the nursery [28]. As only the piglets of the PIGVAC group were vaccinated themselves, all antibodies detected at the end of nursery in the other groups were linked to natural *Salmonella* infection of the piglets. In the two groups only applying sow vaccination, there was no risk of a booster of vaccine induced antibodies through subsequent environmental pathogen contact, which could have impacted the proportion of serological positive animals [29]. Statistical analysis showed a significant effect of *Salmonella* Choleraesuis (SC) vaccination of group PIGVAC. The ELISA OD% and proportion of seroreagents were lower compared to piglets of group UNVAC. Thus, it can be concluded that vaccination against SC (LPS-rough form) also provided protection against ST. This result is in accordance with the results of previous studies that also hypothesized cross-protectivity of a SC vaccine against ST [30–32]. As Salmonella live vaccines are inducing a strong cellular immune response [33], cross-immunity is very likely independent from vaccine-induced antibodies measurable in the used *Salmonella* LPS-ELISA. Especially as the used SC live vaccine contains an attenuated SC rough form, which is characterized by a change in the O-Antigen of the LPS. Thus, in contrast to vaccination with a ST live vaccine containing an attenuated ST strain without changed LPS, the SC vaccine does not prime on LPS and therefore does not have impact on seroreagents in vaccinated animals [34]. This vaccine showed to be an effective tool for *Salmonella* control of advantage in countries where a serology-based *Salmonella* monitoring is performed.

In our study, ELISA OD% values remained at a comparably low level in the three groups SOWVAC-1, SOWVAC-2 and PIGVAC. This means that all vaccination groups (either piglet vaccination or vaccination of the sows) had significantly lower ELISA OD% values than the control group UNVAC at the end of rearing. Hence, it can be concluded that a combination of these two vaccination strategies can be an effective tool to reduce the *Salmonella* seroprevalence. The reduction of seroprevalence in the PIGVAC group could be due to the use of a SC attenuated live vaccine leading to low infection rates, low *Salmonella* shedding and a significant reduction in environmental infection pressure. The following groups SOWVAC-1 and SOWVAC-2 may have benefited from this reduced environmental contamination. It is known that shedding of ingested *Salmonella* and the immune response against this pathogen depend on the *Salmonella* type as well as the ingested bacterial dose [35]. The varying frequencies of environmental samples positive for *Salmonella* after cleaning and disinfection can be explained by the fact that different rooms of the farm were sampled and different degrees of room contamination after cleaning and disinfection could be expected. However, there is a trend towards a decrease in positive environmental samples over time. This could indicate a sustained improvement in cleaning and disinfection management on the farm. However, it is also conceivable that sow vaccination alone would have resulted in a reduction in ELISA OD% values at the end of piglet rearing. Two studies resulted in lower shedding of *Salmonella* in fattening pigs whose mothers received ST vaccination compared to a control group from unvaccinated mothers [20, 36]. Another recent longitudinal study found lower serological values in slaughter pigs from vaccinated sows compared to pigs from unvaccinated sows [13]. If the effect of a sow vaccination is detectable at the time of slaughter, it can be assumed that it is also detectable at the end of piglet rearing. The German *Salmonella* surveillance program is based on a high cut-off value (OD% ≥ 40) to distinguish between negative and positive samples. In our study, a significant influence of vaccination status of either sows or piglets on the serological status of the piglets (positive: OD% ≥ 40 vs. negative: OD% ≤ 40) was found. PIGVAC, SOWVAC-1, and SOWVAC-2 had significantly higher odds of being negative at the end of piglet rearing compared to the UNVAC group. The cut-offs provided for LPS-ELISA tests differ between the manufacturers. In the Idexx Swine Salmonella antibody test kit (Idexx, Westbrook, Maine, US) a sample with an OD% value of more than 10 is considered positive for laboratory diagnostical interpretation. Even based on this lower cut-off, vaccination was found to have an impact on the ELISA OD% values at the end of nursery. While UNVAC piglets had a mean OD% value of 20 at this time, which is positive based on laboratory diagnostical interpretation, the PIGVAC (mean OD% value: 4), SOWVAC-1 (mean OD% value: 4) and SOWVAC-2 (mean OD% value: 10) groups had significantly lower mean OD % values. A significant difference was detectable between SOWVAC-1 and SOWVAC-2 when comparing the ELISA OD% values. The probability of being positive (OD% ≥ 40) at the end of piglet rearing was higher for SOWVAC-2 piglets than for SOWVAC-1 piglets. The longer a vaccination is established in a sow herd, the more stable the herd should be and the less S*almonella* should be introduced from the farrowing farm into the piglet rearing farm. Consequently, this should lead to a lower seroprevalence in the piglet rearing farm over time. Within the study period this effect could not be shown. However, recent studies suggest that it is not possible to extrapolate from the *Salmonella* load of the farrowing farm to the *Salmonella* load of the piglet rearing farm [37]: The study used sock samples to compare *Salmonella* levels in three piggeries with those in downstream piglet rearing farms. The piggery sampled with the lowest *Salmonella* load had the highest load in the downstream piglet rearing farm and vice versa.

This difference between two groups of piglets, both born from vaccinated sows, may have various reasons. On the one hand, as unvaccinated piglets were moved to the piglet rearing farm (after PIGVAC), these piglets were susceptible for *Salmonella* load in the environment. As unvaccinated piglets from vaccinated sows can get reinfected by these environmental *Salmonella*, they might shed to a higher extend and in consequence lead to an increase of environmental pressure and thus to a rise in *Salmonella* antibody levels. It is also known that sows are a reservoir for *Salmonella* and also shed *Salmonella* around farrowing [38]. As in March 2020 still *Salmonella* could be detected in cleaned and disinfected compartments in the rearing farm, it is very likely, that the environmental contamination lead to infection of the non-vaccinated piglets in nursery and consequently to seroconversion at the end of rearing.

### Fattening pigs

The ELISA-OD% values for the four fattening farms show considerable farm-specific differences for the four tested groups and, in part, also diverse trends between the different fattening farms during this longitudinal study (see Fig. 4a–d). No patterns can be derived from these trends. The same applies to the number of serological positive animals (see Fig. 5a–d). This highlights the outstanding influence of the farm management on *Salmonella* control. For example, building conditions (e.g., flooring, materials used in the barn) can have a major impact on the effectiveness of cleaning and disinfection measures and thus on the residual bacterial flora [39]. Another important factor is protection of the barn from invading rodents and birds as potential reservoirs for *Salmonella* [40, 41] which is often difficult to ensure especially in old buildings. Consistent implementation of biosecurity measures (change of boots) and a pig flow that prevents contact between different age groups are further measures with high impact [42]. The varying proportions of seropositive animals in the different fattening farms at the end of finishing phase linked to the sample source of piglets reflect the risk of bias if a general statement is derived from the results of only one farm. For example, while the results of fattener 2 suggest a decrease, the results of fattener 1 suggest an increase of *Salmonella* seroprevalence over the course of the study. Considering the ELISA-OD% values of the slaughter pigs summarizing all fattening farms, a significant influence of the SC vaccination (group PIGVAC) can be shown. The UNVAC, SOWVAC-1, and SOWVAC-2 groups were not significantly different from each other, but all groups showed significantly higher ELISA OD% values than the PIGVAC group. It is likely that piglet vaccination against SC protected piglets against ST infection and made them less susceptible to risk factors promoting *Salmonella* infections compared to pigs from ST vaccinated mothers. Sow vaccination reduces the likelihood of piglet infection during lactation by reducing the sow's shedding of *Salmonella*. Maternal derived antibodies against *Salmonella* are detectable up to eight weeks [43, 44]. A new increase in antibodies a few weeks after the decrease in maternal antibodies is indicative for field infection with *Salmonella* [44]. It can be expected that MDA lasting until the first weeks of nursery reduce colonization by *Salmonella* (18). After this time piglets are considered fully susceptible to *Salmonella* infection, so that the long-lasting effectivity of sow vaccination depends on the elimination of *Salmonella* exposure and further risk factors in the rearing and fattening period.

## Conclusions and recommendations

The pig’s environment as a *Salmonella* reservoir is a high risk factor not only for the fattening pigs but for all upstream production stages, so that the risk for *Salmonella* exposure should be minimized before implementation of a vaccination strategy. While the close cooperative relationship between piglet producer and piglet rearing farm resulted in optimal conditions for studying the vaccination effect until the end of nursery period, the effect of vaccination at the end of fattening in the various downstream fattening farms cannot be evaluated so clearly, because additional factors presumably had an impact on the results. In this project, an optimized cleaning and disinfection protocol followed by vaccination of piglets against SC and gilts/sows against ST resulted in a reduction of *Salmonella*-positive serum samples at the end of nursery. To reduce the *Salmonella* burden and other risk factors on farms detailed instructions specific for each farm should be elaborated taking the individual environmental conditions, feeding strategies and management into account. The quality management principle of "plan-do-check-act" should be continuously applied on farms affected by *Salmonella*. The coordination of all parties involved in the production chain requires target-oriented communication, compliance with measures and assignment of responsibilities down to the level of all persons operating on farm. Finally, consistent implementation and long-term maintenance of the above mentioned measures in all production stages, which may include the vaccination of piglets, are the first prerequisite for a successful *Salmonella* vaccination strategy in gilts and sows, which can only be effective in the medium and long term.

## Data Availability

No datasets were generated or analysed during the current study.

## References

[1] European Food Safety Authority (EFSA). The European Union One Health 2022 Zoonoses report. EFSA J. 2023;21(12):8442. 10.2903/j.efsa.2023.8442 PMC10714251 38089471

[2] Crump JA, Sjölund-Karlsson M, Gordon MA, Parry CM. Epidemiology, clinical presentation, laboratory diagnosis, antimicrobial resistance, and antimicrobial management of invasive salmonella infections. Clin Microbiol Rev. 2015;28(4):901–37. 26180063 10.1128/CMR.00002-15 PMC4503790

[3] Chen HM, Wang Y, Su LH, Chiu CH. Nontyphoid Salmonella infection: microbiology, clinical features, and antimicrobial therapy. Pediatr Neonatol. 2013;54(3):147–52. 23597525 10.1016/j.pedneo.2013.01.010

[4] Arguello H, Alvarez-Ordontez A, Carvajal A, Rubio P, Prieto M. Role of slaughtering in Salmonella spreading and control in pork production. J Food Prot. 2013;76(5):899–911. 23643137 10.4315/0362-028X.JFP-12-404

[5] Smid JH, Van Hoek A, Aarts HJM, Havelaar AH, Heres L, De Jonge R, Pielaat A. Quantifying the sources of Salmonella on dressed carcasses of pigs based on serovar distribution. Meat Sci. 2014;96(4):1425–31. 24398002 10.1016/j.meatsci.2013.12.002

[6] Gomes-Neves E, Antunes P, Tavares A, Themudo P, Cardoso MF, Gärtner F, Costa JM, Peixe L. Salmonella cross-contamination in swine abattoirs in Portugal: carcasses, meat and meat handlers. Int J Food Microbiol. 2012;157(1):82–7. 22607810 10.1016/j.ijfoodmicro.2012.04.015

[7] Correia-Gomes C, Leonard F, Graham D. Description of control programmes for Salmonella in pigs in Europe. Progress to date? J Food Safety. 2021;41(5):e12916.

[8] De Lucia A, Ostanello F. On-farm risk factors associated with Salmonella in pig herds. Large Anim Rev. 2020;26:133–40.

[9] Berends BR, Urlings HAP, Snijders JMA, Van Knapen F. Identification and quantification of risk factors in animal management and transport regarding Salmonella spp. in pigs. Int J Food Microbiol. 1996;30(1–2):37–53. 8856373 10.1016/0168-1605(96)00990-7

[10] Bonardi S. Salmonella in the pork production chain and its impact on human health in the European Union. Epidemiol Infect. 2017;145:1513–26. 28241896 10.1017/S095026881700036X PMC9203350

[11] Correia-Gomes C, Economou T, Mendonça D, Vieira-Pinto M, Niza-Ribeiro J. Assessing risk profiles for Salmonella serotypes in breeding pig operations in Portugal using a Bayesian hierarchical model. BMC Vet Res. 2012;8:226. 23171637 10.1186/1746-6148-8-226 PMC3514327

[12] Roesler U, Heller P, Waldmann KH, Truyen U, Hensel A. Immunization of sows in an integrated pig-breeding herd using a homologous inactivated Salmonella vaccine decreases the prevalence of Salmonella Typhimurium infection in the offspring. J Vet Med B. 2006;53:224–8. 10.1111/j.1439-0450.2006.00951.x 16732880

[13] Peeters L, Dewulf J, Boyen F, Brossé C, Vandersmissen T, Rasschaert G, Heyndrickx M, Cargnel M, Mattheus W, Pasmans F, Haesebrouck F, Maes D. Evaluation of group vaccination of sows and gilts against Salmonella Typhimurium with an attenuated vaccine in subclinically infected pig herds. Prev Vet Med. 2020;182: 104884. 32536448 10.1016/j.prevetmed.2020.104884

[14] De la Cruz ML, Conrado I, Nault A, Perez A, Dominguez L, Alvarez J. Vaccination as a control strategy against Salmonella infection in pigs: a systematic review and meta-analysis of the literature. Res Vet Sci. 2017;114:86–94. 28340428 10.1016/j.rvsc.2017.03.005

[15] Wales AD, Davies RH. Salmonella Vaccination in pigs: a review. Zoonoses Public Health. 2017;64:1–13. 26853216 10.1111/zph.12256

[16] Roesler U, Marg H, Schröder I, Mauer S, Arnold T, Lehmann J, Truyen U, Hensel A. Oral vaccination of pigs with an invasive gyrA-cpxA-rpoB Salmonella Typhimurium mutant. Vaccine. 2004;23(5):595–603. 15542179 10.1016/j.vaccine.2004.07.013

[17] Springer S, Lindner T, Steinbach G, Selbitz H-J. Investigation of the efficacy of a genetically-stabile live Salmonella Typhimurium vaccine for use in swine. Berl Munch Tierarztl. 2001;114(9–10):342–5. 11570173

[18] Casanova-Higes A, Marín-Alcalá CM, Andrés-Barranco S, Cebollada-Solanas A, Alvarez J, Mainar-Jaime RC. Weaned piglets: another factor to be considered for the control of Salmonella infection in breeding pig farms. Vet Res. 2019;50:45. 31215485 10.1186/s13567-019-0666-7 PMC6582532

[19] van der Wolf P, Meijerink M, Libbrecht E, Tacken G, Gijsen E, Lillie-Jaschniski K, Schüller V. Salmonella Typhimurium environmental reduction in a farrow-to-finish pig herd using a live attenuated Salmonella Typhimurium vaccine. Porc Health Manag. 2021;7:43. 10.1186/s40813-021-00222-1 PMC8299633 34301340

[20] Davies R, Gosling B, Wales AD, Smith RP. Use of an attenuated live Salmonella Typhimurium vaccine on three breeding pig units: a longitudinal observational field study. Comp Immuno Microb. 2016;46:7–15. 10.1016/j.cimid.2016.03.005 27260804

[21] Martelli F, Lambert M, Butt P, Cheney T, Tatone FA, Callaby R, Rabie A, Gosling RJ, Fordon S, Crocker G, Davies RH, Smith RP. Evaluation of an enhanced cleaning and disinfection protocol in Salmonella contaminated pig holdings in the United Kingdom. PLoS ONE. 2017;12(6): e0178897. 28594930 10.1371/journal.pone.0178897 PMC5464571

[22] De Ridder L, Maes D, Dewulf J, Pasmans F, Boyen F, Haesebrouck F, Meroc E, Butaye P, Van der Stede Y. Evaluation of three intervention strategies to reduce the transmission of Salmonella Typhimurium in pigs. Vet J. 2013;197(3):613–8. 23680264 10.1016/j.tvjl.2013.03.026

[23] Andres VM, Davies RH. Biosecurity measures to control Salmonella and other infectious agents in pig farms: a review. Compr Rev Food Sci F. 2015;14(4):317–35.

[24] Bernad-Roche M, Casanova-Higes A, Marín-Alcalá CM, Cebollada-Solanas A, Mainar-Jaime RC. Salmonella infection in nursery piglets and its role in the spread of Salmonellosis to further production periods. Pathogens. 2021;10(2):123. 33504097 10.3390/pathogens10020123 PMC7911055

[25] Robertson M. Survival of S. typhimurium in floor dust-a possible reservoir of infection in institutions. Public Health. 1972;87(1):39–45. 4605194 10.1016/s0033-3506(72)80034-9

[26] Schilling T, Hoelzle K, Philipp W, Hoelzle LE. Survival of Salmonella Typhimurium, Listeria monocytogenes, and ESBL carrying *Escherichia coli* in stored anaerobic biogas digestates in relation to different biogas input materials and storage temperatures. Agriculture. 2022;12(1):67.

[27] Cevallos-Almeida M, Fablet C, Houdayer C, Dorenlor V, Eono F, Denis M, Kerouanton A. Longitudinal study describing time to Salmonella seroconversion in piglets on three farrow-to-finish farms. Vet Rec Open. 2019;6(1): e000287. 31673373 10.1136/vetreco-2018-000287 PMC6802978

[28] Kranker S, Alban L, Boes J, Dahl J. Longitudinal study of Salmonella enterica Serotype Typhimurium infection in three Danish Farrow-to-Finish swine herds. J Clin Microbiol. 2003;41(6):2282–8. 12791837 10.1128/JCM.41.6.2282-2288.2003 PMC156512

[29] Theuß T, Ueberham E, Lehmann J, Lindner T, Springer S. Immunogenic potential of a Salmonella Typhimurium live vaccine for pigs against monophasic Salmonella Typhimurium DT 193. BMC Vet Rec. 2017;13:343. 10.1186/s12917-017-1271-5 PMC5693801 29149900

[30] Charles SD, Abraham AS, Trigo ET, Jones GF. Reduced shedding and clinical signs of Salmonella Typhimurium in nursery pigs vaccinated with a Salmonella Choleraesuis vaccine. J Swine Health Prod. 2000;8(3):107–12.

[31] Maes D, Gibson K, Trigo E, Szaszák Á, Grass J, Carlson A, Blaha T. Evaluation of cross-protection afforded by a Salmonella Choleraesuis vaccine against Salmonella infections in pigs under field conditions. Berl Munch Tierarztl. 2001;114(9–10):339–41. 11570172

[32] Moura EAGdO, Silva DGd, Turco CH, Sanches TVC, Storino GY, Almeida HMdS, Mechler-Dreibi ML, Rabelo IP, Sonalio K, Oliveira LGd. Salmonella bacterin vaccination decreases shedding and colonization of Salmonella Typhimurium in pigs. Microorganisms. 2021;9(6):1163. 10.3390/microorganisms9061163 PMC8226585 34071310

[33] Schmidt S, Sassu EL, Vatzia E, Pierron A, Lagler J, Mair KH, et al. Vaccination and Infection of Swine with Salmonella Typhimurium Induces a Systemic and Local Multifunctional CD4+ T-Cell Response. Front Immunol. 2020;11: 603089. 33584671 10.3389/fimmu.2020.603089 PMC7874209

[34] Peeters L, Dewulf J, Boyen F, Brossé C, Vandersmissen T, Rasschaert G, Heyndrickx M, Cargnel M, Pasmans F, Maes D. Effects of attenuated vaccine protocols against Salmonella Typhimurium on Salmonella serology in subclinically infected pig herds. Vet J. 2019;249:67–72. 31239168 10.1016/j.tvjl.2019.05.008

[35] Ivanek R, Österberg J, Gautam R, Sternberg LS. Salmonella fecal shedding and immune responses are dose-and serotype-dependent in pigs. PLoS ONE. 2012;7(4): e34660. 22523553 10.1371/journal.pone.0034660 PMC3327719

[36] Smith RP, Andres V, Martelli F, Gosling B, Marco-Jimenez F, Vaughan K, Tchorzewska M, Davies R. Maternal vaccination as a Salmonella Typhimurium reduction strategy on pig farms. J Appl Microbiol. 2018;124(1):274–85. 29024207 10.1111/jam.13609

[37] Hollmann I, Lingens JB, Wilke V, Homann C, Teich K, Buch J, Chuppava B, Visscher C. Epidemiological study on Salmonella prevalence in sow herds using direct and indirect detection methods. Microorganisms. 2022;10(8):1532. 36013949 10.3390/microorganisms10081532 PMC9413226

[38] Larivière-Gauthier G, Thibodeau A, Letellier A, Yergeau É, Fravalo P. Reduction of Salmonella shedding by sows during gestation in relation to its fecal microbiome. Front Microbiol. 2017;8:2219. 29209285 10.3389/fmicb.2017.02219 PMC5701629

[39] Funk J, Gebreyes WA. Risk factors associated with Salmonella prevalence on swine farms. J Swine Health Prod. 2004;12(5):246–51.

[40] Nollet N, Maes D, De Zutter L, Duchateau L, Houf K, Huysmans K, Imberechts H, Geers R, de Kruif A, Van Hoof J. Risk factors for the herd-level bacteriologic prevalence of Salmonella in Belgian slaughter pigs. Prev Vet Med. 2004;65(1–2):63–75. 15454327 10.1016/j.prevetmed.2004.06.009

[41] Vico JP, Rol I, Garrido V, San Román B, Grilló MJ, Mainar-Jaime RC. Salmonellosis in finishing pigs in Spain: prevalence, antimicrobial agent susceptibilities, and risk factor analysis. J Food Prot. 2011;74(7):1070–8. 21740708 10.4315/0362-028X.JFP-10-515

[42] Giraldo-Cardona JP, Gualdrón-Ramírez D, Chamorro-Tobar I, Pulido-Villamarín A, Santamaría-Durán N, Castañeda-Salazar R, Castaneda-Salazar R, Zambrano-Moreno C, Carrascal-Camacho AK. Salmonella spp. prevalence, antimicrobial resistance and risk factor determination in Colombian swine farms. Pesq Vet Bras. 2019;39(10):816–22.

[43] Beloeil PA, Chauvin C, Proux K, Rose N, Queguiner S, Eveno E, Houdayer C, Rose V, Fravalo P, Madec F. Longitudinal serological responses to Salmonella enterica of growing pigs in a subclinically infected herd. Prev Vet Med. 2003;60(3):207–26. 12900159 10.1016/s0167-5877(03)00126-0

[44] Proux K, Houdayer C, Humbert F, Cariolet R, Rose V, Eveno E, Madec F. Development of a complete ELISA using Salmonella lipopolysaccharides of various serogroups allowing to detect all infected pigs. Vet Res. 2000;31(5):481–90. 11050743 10.1051/vetres:2000134

